# Case report: Severe non-pigmenting fixed drug eruption showing general symptoms caused by chondroitin sulfate sodium

**DOI:** 10.3389/fmed.2022.1041492

**Published:** 2022-10-28

**Authors:** Haruhiko Otsuka, Takeshi Fukumoto, Masahiro Oka, Chikako Nishigori

**Affiliations:** ^1^Division of Dermatology, Department of Internal Related, Kobe University Graduate School of Medicine, Kobe, Japan; ^2^Department of Dermatology, Kita-Harima Medical Center, Ono, Japan

**Keywords:** non-pigmenting fixed drug eruption, general symptoms, chondroitin sulfate sodium, histopathological examination, melanophages

## Abstract

Non-pigmenting fixed drug eruption (NPFDE) is a subtype of fixed drug eruption (FDE) in which repeated eruptions occur at the same site. Clinically, NPFDE disappears without pigmentation changes; however, it sometimes causes fever or arthralgia. Its histopathological characteristics reportedly include infiltrations of CD8-positive T cells with a paucity of melanocytes as compared to FDE. We present the first case of severe NPFDE exhibiting general symptoms caused by chondroitin sulfate sodium. The patient was a 44-year-old man. Intravenous injection of chondroitin sulfate sodium caused erythema in the affected area. A histopathological examination of the biopsy tissue revealed infiltration of CD3-positive lymphocytes (both CD4-positive and CD8-positive lymphocytes) into the epidermis, minimal liquefaction degeneration in the basal layer of the epidermis, and few dermal melanophages, which may be responsible for non-pigmentation.

Non-pigmenting fixed drug eruption (NPFDE) is a rare subtype of fixed drug eruption (FDE) characterized by repeated eruptions occurring at the same site (1). Clinically, NPFDE resolves without pigmentation changes; however, it sometimes causes fever or arthralgia (1, 2). Herein, we present a case of severe NPFDE exhibiting general symptoms caused by chondroitin sulfate sodium. To the best of our knowledge, this is the first case of its kind. Moreover, the histopathological examination of the biopsy specimen from our patient showed NPFDE characteristics that possibly caused the clinical phenotype (1, 3, 4).

The patient was a 44-year-old male involved in a traffic accident that resulted in neck pain. He was treated with an intravenous injection of a mixed agent containing

chondroitin sulfate sodium and sodium salicylate three times every second day. Four days after three courses of treatment (total chondroitin sulfate sodium = 600 mg), the patient developed erythematous skin eruptions with clear boundaries, which started on the right knee and subsequently spread all over the body except for the upper back and the ends of the extremities. Additionally, the patient had fever and arthralgia (Figures 1a–e).

**FIGURE 1:**
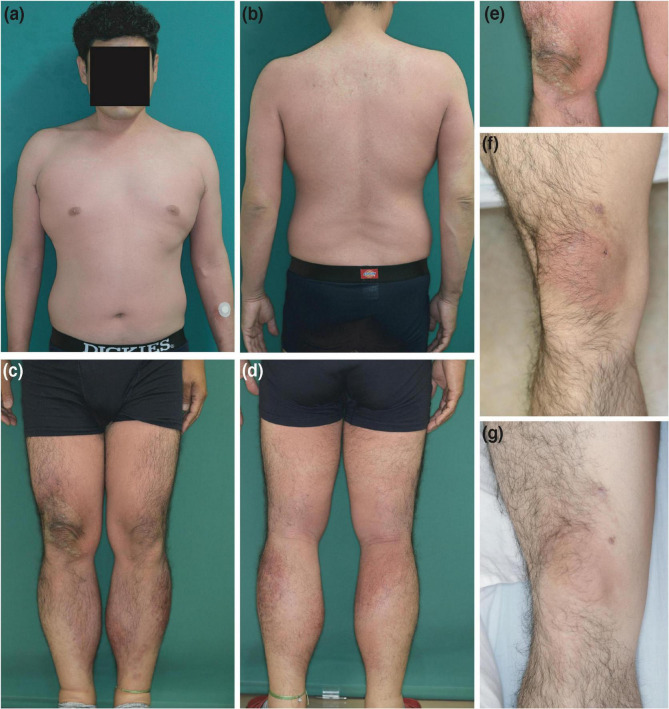
Clinical features. **(a–e)** Erythematous skin eruptions that first appeared on the right knee and subsequently spread all over the body, except for the upper back and the ends of the extremities, at the first consultation. **(f)** Clearly demarcated erythema on the right knee only after the intravenous injection (as is) with the mixed agent containing chondroitin sulfate sodium and sodium salicylate. **(g)** Clearly demarcated erythema on the right knee after intravenous injection with chondroitin sulfate sodium (as is).

The histopathological examination revealed infiltration of CD3-positive lymphocytes (both CD4-positive and CD8-positive lymphocytes) into the epidermis. In addition, there was minimal liquefaction degeneration in the basal layer of the epidermis and few dermal melanophages (Figures 2a–e). Furthermore, Melan-A staining did not reveal a paucity of melanocytes, and Fontana-Masson staining did not show increased staining (Figures 2f,h). Additionally, myeloperoxidase staining of the cell infiltrations was negative (Figure 2i), and eosinophils were observed around the blood vessels in the dermis (Figures 2a,b).

**FIGURE 2:**
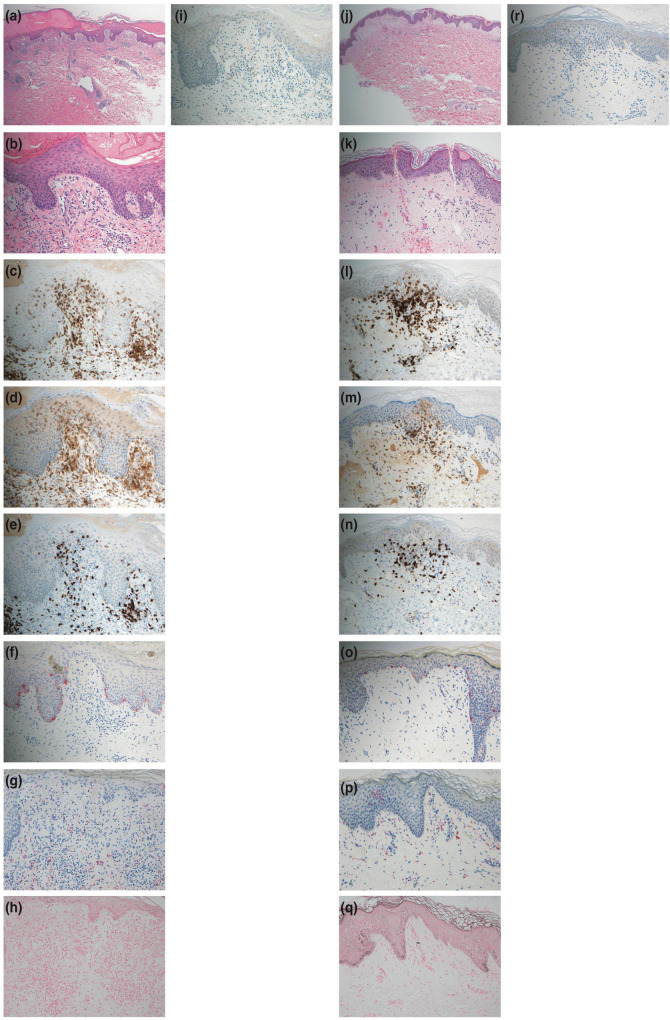
Histopathological features. **(a,b)** Hematoxylin-eosin staining of a skin biopsy specimen from the right knee at the first consultation showing minimal liquefaction degeneration in the basal layer of the epidermis. Also, lymphocytes and eosinophils are observed around the blood vessels of the dermis [panel **(a)** × 40, panel **(b)** × 200]. **(c)** CD3 staining showing positive results in the cell infiltrations (× 200). **(d)** CD4 staining showing positive results in the cell infiltrations (× 200). **(e)** CD8 staining showing positive results in the cell infiltrations (× 200). **(f)** Melan-A staining showing positive results in melanocytes (× 200). **(g)** Few melanophages in the dermis with CD68-positive staining (× 200). **(h)** Fontana-Masson staining showing no increased staining (× 200). **(i)** Myeloperoxidase staining showing negative results in the cell infiltrations (× 200). **(j,k)** Hematoxylin-eosin staining of a skin biopsy specimen from the right knee after the challenge test by intravenous injection (as is) with the mixed agent containing chondroitin sulfate sodium and sodium salicylate showing minimal liquefaction degeneration in the basal layer of the epidermis. Also, lymphocytes and eosinophils are observed around the blood vessels of the dermis [panel **(j)** × 40, panel **(k)** × 200)]. **(l)** CD3 staining showing positive results in the cell infiltrations (× 200). **(m)** CD4 staining showing positive results in the cell infiltrations (× 200). **(n)** CD8 staining showing positive results in the cell infiltrations (× 200). **(o)** Melan-A staining showing positive results in melanocytes (× 200). **(p)** Few melanophages in the dermis with CD68-positive staining (× 200). **(q)** Fontana-Masson staining showing no increased staining (× 200). **(r)** Myeloperoxidase staining showing negative results in the cell infiltrations (× 200).

The mixed agent treatment (containing chondroitin sulfate sodium and sodium salicylate) was discontinued, and the patient received topical clobetasol propionate and an oral antihistamine, which alleviated his skin eruptions within a week, leaving no pigmentation.

The patient provided written informed consent to undergo further testing to isolate the cause of the erythema after we excluded viral etiologies. A patch test was performed on the patient’s upper back (unaffected skin) and the right knee (affected skin), where the erythema first developed, to check for any reaction to the mixed agent containing chondroitin sulfate sodium and sodium salicylate. Both sites were negative. Additionally, the results for the prick and intracutaneous tests at the left forearm were negative. While intravenous injection (1%; 2 mg of chondroitin sulfate sodium) with the mixed agent elicited a negative response, the intravenous injection (as is; 200 mg of chondroitin sulfate sodium) with the mixed agent led to erythema recurrence without pigmentation in the right knee alone, where marked erythema was initially noted (Figure 1f) after 24 h, and without fever and arthralgia. Histopathological examination of the erythematous lesion revealed similar characteristics to those noted previously (Figures 2j–r). Consistent with the clinical images of the knee, histopathological examinations showed hyperkeratosis, potentially caused by mechanical stress.

Subsequently, the effects of chondroitin sulfate sodium and sodium salicylate were independently examined. The results of patch and prick tests on the upper back and right knee with the individual components were negative. No changes occurred after intravenous injections with sodium salicylate (1% and as is) and chondroitin sulfate sodium (1%). However, intravenous injection with chondroitin sulfate sodium (as is) caused erythema recurrence without pigmentation in the right knee alone, similar to the response of the mixed agent (as is) (Figure 1g). Therefore, the patient was diagnosed with NPFDE caused by chondroitin sulfate sodium.

This report emphasizes two critical learning points. First, this was the first case of severe NPFDE exhibiting general symptoms caused by chondroitin sulfate sodium. Second, the NPFDE lesion was characterized by infiltration comprising of CD4-positive and CD8-positive T cells, minimal liquefaction degeneration in the basal layer of the epidermis, and few dermal melanophages. A previous study reported increased CD8-positive T cells in an NPFDE lesion (2); however, in our patient, both CD4- and CD8-positive T cells were present. Therefore, we hypothesized that these CD4-positive T cells might be regulatory T cells, which predominantly infiltrate the lesion, resulting in lesser liquefaction degeneration and a rapid resolution of inflammation. However, further studies are needed to confirm this.

Fixed drug eruption (FDE) is normally characterized by liquefaction degeneration in the basal layer and dermal melanophages, leading to clinical pigmentation (5). However, in our patient, the liquefaction degeneration in the basal layer was minimal, and the number of melanophages in the dermis was limited, despite excessive intraepidermal inflammatory cell infiltration. This can be attributed to the absence of melanophages in the dermis, due to minimal liquefaction degeneration, and the presence of a majority of the CD4-positive and CD8-positive T cells in the epidermis and not the basal layer. As previously reported, these factors may be responsible for the lack of pigmentation (5).

Our report has several limitations. First, our observation is based on a single case. Second, our patient did not react to the patch test, even in the affected area. Andrade et al. (4) have recommended performing the patch test a few weeks to a few months after resolution due to a potential refractory period, which may explain this result (4, 6). Additionally, they reported that less than half the patch tests were reactive, even in the affected area because of some potential reasons such as penetration rate and molecular size of drugs, features of vehicles, and lack of systemic transformation for immune response activation (4, 6). In these cases, oral challenge and intravenous injection of culprit drugs, epidermal thinning (e.g., tape stripping), increased concentration of drugs, and prolonged duration of occlusion may be useful (4, 6).

Nonetheless, we report a rare, severe case of NPFDE showing general symptoms, characterized by minimal liquefaction degeneration with few dermal melanophages and increased CD4-positive and CD8-positive T cells. Chondroitin sulfate sodium is a dietary supplement, commonly used to treat osteoarthritis (7). Therefore, this report may serve as a cautionary case, urging clinicians to consider the risk of NPFDE in any case of erythema following chondroitin sulfate sodium administration.

## Data availability statement

The original contributions presented in this study are included in the article/supplementary material, further inquiries can be directed to the corresponding author.

## Ethics statement

The patient has given informed consent for the publication of this report.

## Author contributions

HO and TF designed the study and contributed to data collection. All authors wrote the manuscript, read, and approved the final manuscript.

## References

[1] Fukuda R Ouchi T Hirai I Funakoshi T Honda A Tanese K Non-pigmenting fixed drug eruption with mixed features of acute generalized exanthematous pustulosis induced by pseudoephedrine: a case report. *Contact Dermatitis.* (2017) 77:123–6. 10.1111/cod.12754 28703347

[2] Mizukawa Y Shiohara T. Nonpigmenting fixed drug eruption as a possible abortive variant of toxic epidermal necrolysis: immunohistochemical and serum cytokine analyses. *Clin Exp Dermatol.* (2010) 35:493–7. 10.1111/j.1365-2230.2009.03622.x 19874369

[3] Shiohara T Ushigome Y Kano Y Takahashi R. Crucial role of viral reactivation in the development of severe drug eruptions: a comprehensive review. *Clin Rev Allergy Immunol.* (2015) 49:192–202. 10.1007/s12016-014-8421-3 24736996

[4] Andrade P Brinca A Goncalo M. Patch testing in fixed drug eruptions–a 20-year review. *Contact Dermatitis.* (2011) 65:195–201. 10.1111/j.1600-0536.2011.01946.x 21702758

[5] Cheraghlou S Levy LL. Fixed drug eruptions, bullous drug eruptions, and lichenoid drug eruptions. *Clin Dermatol.* (2020) 38:679–92. 33341201 10.1016/j.clindermatol.2020.06.010

[6] Woodruff CM Botto N. The role of patch testing in evaluating delayed hypersensitivity reactions to medications. *Clin Rev Allergy Immunol.* (2022) 62:548–61. 35113364 10.1007/s12016-022-08924-2 PMC9156465

[7] Schiraldi C Cimini D De Rosa M. Production of chondroitin sulfate and chondroitin. *Appl Microbiol Biotechnol.* (2010) 87:1209–20. 20521042 10.1007/s00253-010-2677-1

